# Real-world waitlist randomised controlled trial of gameChange VR to treat severe agoraphobic avoidance in patients with psychosis: a study protocol

**DOI:** 10.1136/bmjopen-2025-104636

**Published:** 2025-08-16

**Authors:** Daniel Freeman, Julia Jones, Eloise Prouten, John Sainsbury, Anthony Morrison, Kate Chapman, Emma Cousins, Victoria Altoft, Heather Peel, Thomas Kabir, Jack Myrick, Aitor Rovira, Natalie Rouse, Felicity Waite, Sinéad Lambe, José Leal, Ly-Mee Yu

**Affiliations:** 1 Department of Experimental Psychology, University of Oxford, Oxford, UK; 2 Oxford Health NHS Foundation Trust, Oxford, UK; 3 Black Country Healthcare NHS Foundation Trust, Wolverhampton, UK; 4 Greater Manchester Mental Health NHS Foundation Trust, Manchester, UK; 5 Avon and Wiltshire Mental Health Partnership NHS Trust, Bath, UK; 6 Cornwall Partnership NHS Foundation Trust, Bodmin, UK; 7 Humber Teaching NHS Foundation Trust, Willerby, UK; 8 Department of Psychiatry, University of Oxford, Oxford, UK; 9 Health Economics Research Centre, Nuffield Department of Primary Care Health Sciences, University of Oxford, Oxford, UK; 10 Department of Primary Care Health Sciences, University of Oxford, Oxford, UK

**Keywords:** Digital Technology, Adult psychiatry, Anxiety disorders, Schizophrenia & psychotic disorders, Randomized Controlled Trial

## Abstract

**Introduction:**

Many people with psychosis find the world very frightening. It can be difficult for them to do everyday things—for example, walking down a busy street, travelling on a bus or going to the shops. Sometimes, the fears are so great that individuals rarely leave their homes. gameChange virtual reality therapy is designed to reduce this agoraphobic avoidance. In gameChange, users practise going into computerised immersive versions of ordinary situations. A virtual therapist guides users through the programme. A mental health worker also supports people. People normally do six sessions of gameChange, but now they can do more as headsets can be left with many people. We originally tested gameChange with 346 patients with psychosis. People saw a significant reduction in their fears. People with the most severe problems made the biggest improvements. This led to gameChange receiving National Institute for Health and Care Excellence (NICE) Early Value Assessment (EVA) approval for its use with patients with psychosis who have severe agoraphobic avoidance. NICE EVA approval is conditional on further evidence generation. We aim to carry out a real-world trial of gameChange used in the NHS. The overall aim is to gather evidence on the four essential areas (clinical benefits on agoraphobia, level of engagement and adherence, healthcare resource use, adverse effects) and the two further supporting areas (health-related quality of life, generalisability) identified in the NICE evidence generation plan for gameChange.

**Methods and analysis:**

200 patients with psychosis and severe agoraphobic avoidance will be randomised (1:1) to receive gameChange in addition to treatment as usual (TAU) or to a waitlist control group receiving TAU. Assessments will be conducted blind to group allocation at baseline, 8 weeks (end of treatment) and 26 weeks (follow-up). The trial will be embedded in services in at least seven National Health Service (NHS) trusts across England. The primary outcome is agoraphobic avoidance at 26 weeks assessed with the Oxford Agoraphobic Avoidance Scale. The secondary clinical outcomes are agoraphobic distress, paranoia and social contacts. There will be tests of moderation of the main clinical outcome. Treatment acceptability, adverse effects and cost-effectiveness will also be assessed. The target estimand is the treatment policy estimand and all primary and secondary analyses will be carried out incorporating data from all participants including those who do not complete treatment.

**Ethics and dissemination:**

The trial has received ethical approval from the NHS Health Research Authority and Health and Care Research Wales (25/WA/0081). A key output will be the evidence needed for a NICE guidance update on gameChange and a clear recommendation concerning future routine use in the NHS.

**Trial registration number:**

ISRCTN79060696.

**Strengths and limitations of this study**

- A strength of this randomised controlled trial of gameChange virtual reality is that it is designed to collect information across multiple domains: clinical efficacy, level of engagement and adherence, healthcare resource use, adverse effects, health-related quality of life and generalisability.
- To enhance the generalisability of the results, the trial will be embedded in multiple psychosis services across the country.
- The trial will not be able to determine the components of gameChange that lead to clinical benefits.
- The trial is only powered to detect moderate improvements with treatment in agoraphobic avoidance.

## Introduction

 Psychotic conditions—non-affective or affective—are known as severe mental health conditions. They can cause considerable personal distress to patients and their families. The NHS Long Term Plan^1^ has committed to improving access to evidence-based psychological therapies for people diagnosed with severe mental health disorders.

For many patients diagnosed with psychosis, everyday situations can become anxiety-provoking. Patients may fear, for example, negative judgements, observation, embarrassment, failure, rejection, panicking, deliberate social or physical harm from others, or being unable to cope with voices. The end result is that patients can avoid, or find intensely anxiety-provoking, everyday situations such as walking down the street, going to a local shop or getting on a bus. Patients may find it difficult to even leave their home. In effect, individuals are experiencing symptoms of agoraphobia. We view agoraphobic avoidance of everyday situations as a final common pathway for a variety of fears experienced by many patients with psychosis. In a survey of 1809 patients with non-affective psychosis attending NHS mental health services, we found anxious avoidance at agoraphobic levels for 64.5% of patients.^2^

We harnessed a consumer technology, virtual reality (VR), to help people in this patient group who were experiencing such difficulties. In our automated psychological therapy, gameChange, patients can practise overcoming their difficulties in simulations of the everyday situations that they fear. The automation of therapy in gameChange means that it can be supported by a range of NHS staff and require less of their time. gameChange is a cognitive treatment that aims for patients to relearn safety by testing their fear expectations. Within the VR environments, a virtual coach guides the person through the treatment. When first entering VR, the patient goes into the coach’s virtual office and is guided in how to use VR. At the beginning of the first session, the virtual coach explains the rationale behind the treatment, and the participant selects one of the six VR scenarios (eg, leaving the home, getting on a bus, ordering a coffee or tea in a café). Each scenario comprises five levels of difficulty and participants work their way through the tasks in each level.

Two outcome papers have been reported from a randomised controlled clinical trial with 346 patients with psychosis.^3 4^ These show that for any patient with psychosis who has agoraphobia symptoms, there is a significant reduction in fears at the end of treatment. However, a moderation analysis revealed that it was patients with severe agoraphobia and the highest overall mental health symptoms who had the largest gains both post-treatment and 6 months later. It is notable that, 6 months post-treatment, the 6-week gameChange intervention in patients with severe agoraphobia reduced agoraphobia to a large extent (effect size=0.8), reduced paranoia to a moderate extent (effect size=0.4) and improved quality of life to a moderate extent (effect size=0.5). No patient serious adverse events (SAEs) during the trial were linked with gameChange. A peer-led qualitative evaluation reported great enthusiasm for the approach from trial participants.^5^ A paper on satisfaction and side effects showed high satisfaction ratings and low occurrence of side effects that did not affect treatment outcomes or uptake.^6^ And a health economic evaluation^7^ indicated that gameChange may lower costs from an NHS and social care perspective and, by reducing informal caring, from a wider societal perspective too. The analysis also suggested that gameChange may be cost-effective against National Institute for Health and Care Excellence (NICE) thresholds for value per quality-adjusted life-year (QALY) gained.

On the basis of the randomised controlled trial results, gameChange obtained NICE Early Value Assessment (EVA) for medtech approval for use in the NHS: ‘gameChangeVR (a VR technology) can be used in the NHS while more evidence is generated, to treat severe agoraphobic avoidance in people with psychosis aged 16 and over. It should be used with the support of a mental health professional’. The approval is for use for patients diagnosed with psychosis who have severe agoraphobic avoidance (ie, the group that the moderation analysis indicates benefits most). NICE EVA approval is conditional on further evidence generation. The current project is designed to address the evidence gaps identified in the NICE EVA of gameChange.

### Aim and objective

The overall aim is to gather evidence on the four essential areas (clinical benefits on agoraphobia, level of engagement and adherence, healthcare resource use, adverse effects) and the two further supporting areas (health-related quality of life, generalisability) identified in the NICE evidence generation plan for gameChange.

#### Clinical benefits

Primary clinical question: For NHS patients diagnosed with psychosis and having severe agoraphobia, does gameChange when added to treatment as usual (TAU), compared with TAU, lead to a reduction in severe agoraphobic avoidance at 6 months?

Secondary questions:

Compared with usual care, does the addition of gameChange lead to reductions in paranoia?

Compared with usual care, does the addition of gameChange increase the number of social contacts outside the home?

What are the recovery and relapse rates in agoraphobic avoidance in each arm of the trial (as assessed by the bands of the Oxford Agoraphobic Avoidance Scale (O-AS), which is the primary outcome measure)?

#### Level of engagement and adherence

Primary question: What are the levels of engagement and adherence with gameChange?

#### Healthcare resource use

Primary question: What are the total costs of gameChange?

Secondary question: What are the standard care costs associated with gameChange compared with standard care alone?

#### Adverse effects

Primary question: Are there any SAEs linked to the use of gameChange?

Secondary question: What proportion of patients have side effects from gameChange and do these interfere with adherence or outcomes?

#### Health-related quality of life

Primary question: Is gameChange cost-effective?

Secondary question: Compared with usual care, does the addition of gameChange lead to improvements in quality of life?

#### Generalisability

Primary question: Is gameChange an acceptable treatment for patients?

Secondary questions:

Are gameChange treatment outcomes moderated by socio-demographic or clinical factors (eg,age, gender, ethnicity, location, diagnosis)?

Do levels of engagement and adherence vary by sociodemographic or clinical factors?

## Methods and analysis

### Trial design and flow chart

**Figure 1:**
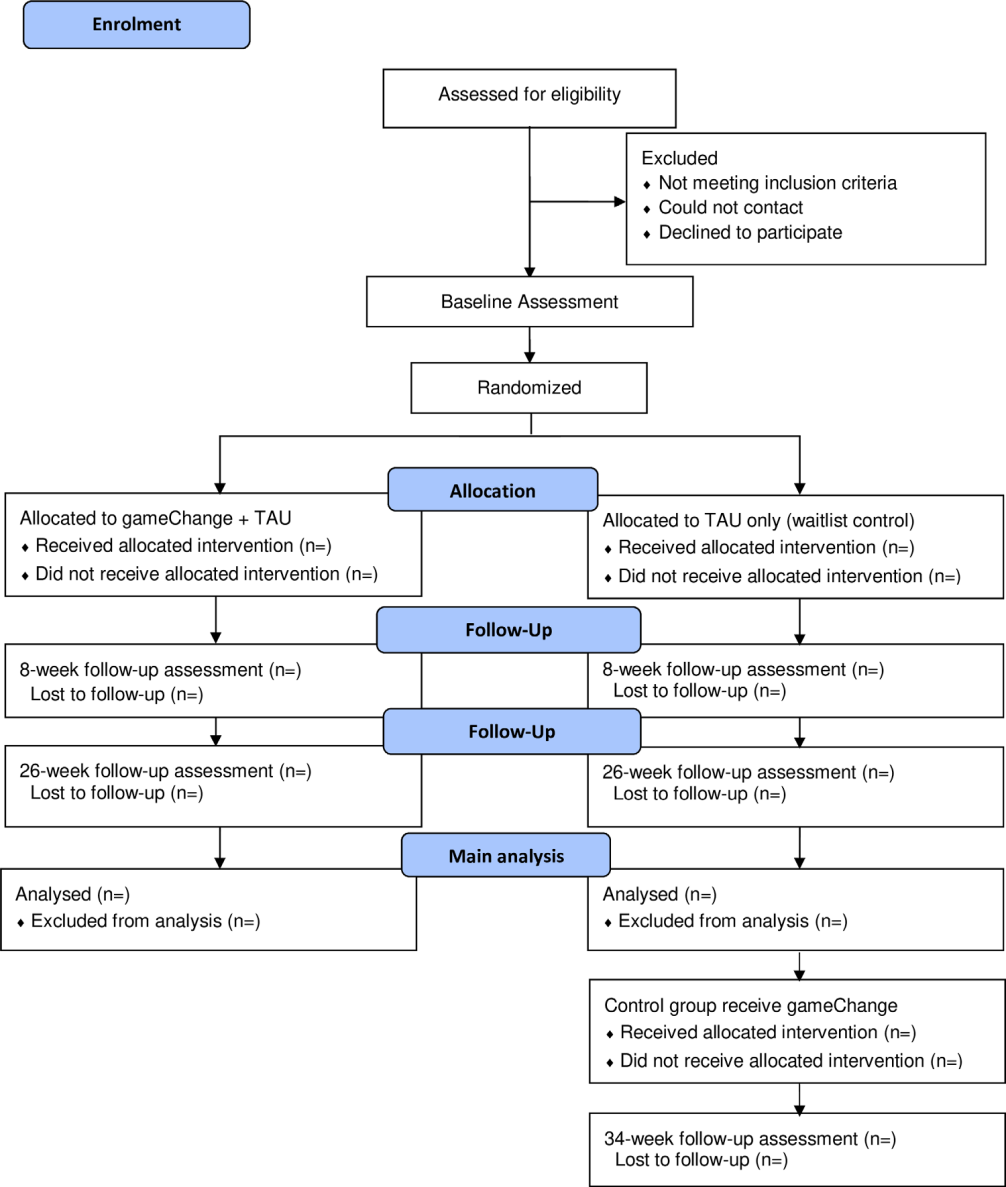
Trial flow diagram. TAU, treatment as usual.

The design is a multicentre two-arm waitlist randomised controlled trial testing the addition of gameChange to TAU against TAU. We wish to determine the ‘in toto’ benefits of the VR intervention when added to standard care (ie, the total effect for patients if implemented in the NHS). 200 patients will be randomised to one of two conditions: gameChange (added to TAU) or a waitlist control group (continuing to receive TAU). gameChange will be provided for 8 weeks with a staff member. Assessments will be conducted blind to group allocation at 0, 8 and 26 weeks. The waitlist control group will also be assessed after treatment (34 weeks). A summary of the trial design can be seen in figure 1. The trial will take place in seven sites: Avon and Wiltshire Mental Health Partnership NHS Trust; Black Country Healthcare NHS Foundation Trust; Cornwall Partnership NHS Foundation Trust; Gloucestershire Health & Care NHS Foundation Trust; Greater Manchester Mental Health NHS Foundation Trust; Humber Teaching NHS Foundation Trust; and Oxford Health NHS Foundation Trust. The trial is prospectively registered with the ISRCTN registry: ISRCTN79060696. The University of Oxford is the trial sponsor. There will be a data monitoring and ethics committee (DMEC).

### Randomisation, blinding and code-breaking

Patients will be randomised once they have completed the baseline assessment. Randomisation, using an online system from Sealed Envelope (https://www.sealedenvelope.com/), will use a permuted blocks algorithm, with randomly varying block size, stratified by site.

The research assessors will be blind to group allocation, but the participants and staff members present will not be (they cannot be blinded to whether a psychological intervention is delivered or not). The gameChange staff deliverers (or trial co-ordinators) will inform patients of the randomisation outcome via telephone, to ensure the research assessors remain blinded to group allocation. Precautionary strategies to prevent unblinding of allocation include: the staff member and assessor considering room use and booking arrangements; patients being reminded by the assessor not to talk about their allocation result; and, after the initial assessment, the assessor not looking at the patient’s clinical notes. If an allocation is revealed between assessment sessions, this is logged by the trial coordinator and whenever possible, reblinding will occur using another assessor.

### Participants

The principal method of recruitment will be via the relevant clinical teams in the participating mental health services (eg, adult community mental health teams; early intervention services, inpatient units, mental health and well-being hubs, commissioned Voluntary, Community and Social Enterprise (VCSE). Recruitment can also be via research registers, where patients have given consent to be contacted about studies. As this is a real-world evaluation, clinical teams will be encouraged to use the Oxford Agoraphobia Avoidance Scale (O-AS)^8^ as part of routine assessment of patients with psychosis. This would identify people for whom gameChange may well be a suitable fit. It will also be the case that staff deliverers in the clinical teams, or Research Delivery Network research assistants embedded in the clinical teams, would be able to check eligibility against clinical notes.

Informed consent (see online supplemental materials) will be obtained from participants. This will be after provision of the patient information sheet (see online supplemental materials) to the patient, opportunities to ask questions, and at least 24 hours to decide. Our Lived Experience Advisory Panel (LEAP) has also emphasised the importance of patients in participating trusts being able to self-refer to the trial. This will minimise the chances that patients are overlooked by clinical teams or because their clinician was not present at a referral meeting. Hence, we will also advertise the study to patients via posters and leaflets in NHS buildings. However, in all instances, we will seek to confirm that a member of the clinical team gives approval for a patient to enter the trial and to complete the necessary check of eligibility and risk status. Written informed consent will be obtained from all participants.

The inclusion criteria are: participant is willing and able to give informed consent for participation in the trial; aged 16 years or older; attending NHS or commissioned VCSE mental health services; clinical diagnosis of schizophrenia spectrum psychosis or an affective diagnosis with psychotic symptoms; severe agoraphobic avoidance as assessed by the O-AS (score of 6 or above)^8^ and wanting help for that difficulty.

The exclusion criteria are: photosensitive epilepsy; in forensic settings or psychiatric intensive care unit; substantial difficulties in understanding or answering trial assessment questions; a participant may also not enter the trial if there is another factor (eg, current active suicidal plans that need to be the focus of intervention), which, in the judgement of the investigator, would preclude the participant from providing informed consent or from safely engaging with the trial procedures. Reason for exclusion will be recorded.

### Assessments

The primary outcome is agoraphobic avoidance assessed by the O-AS.^8^

The secondary outcomes are: agoraphobic distress (O-AS)^8^; paranoia (Revised Green *et al* Paranoid Thoughts Scale)^9^; number of social contacts (Social Contact Assessment).^10^

Treatment acceptability will be measured postintervention by the Theoretical Framework of Acceptability questionnaire^11^ and side effects by the Oxford—VR Side Effects Checklist.^6^ These data are only collected after people receive gameChange VR therapy. These assessments will be administered via an electronic link (or paper copy) provided by the treatment deliverer.

Basic sociodemographic and clinical information will be collected for all participants (age, gender, ethnicity, main language, clinical diagnosis, socioeconomic status, psychiatric medication).

The health economic evaluation will use the: EQ-5D five-level (5L),^12^ 20-item Recovering Quality of Life (ReQol-20)^13^ and adapted version of the Client Service Receipt Inventory (CSRI).^7 14^

### The psychological intervention: gameChange VR

The treatment being tested is gameChange, which is UKCA/CE marked and Digital Technology Assessment Criteria approval is in place. It has NICE EVA approval for use in the NHS with patients with psychosis experiencing severe agoraphobic avoidance. The gameChange software application is composed of a set of virtual environments, including different scenes created using 3D models, ambient audio and 3D virtual characters, with animations and speech. The environments are driven by source code which handles the logic of the programme, the behaviour of the computer characters, as well as the user interaction and data storage. gameChange is certified as a VR application for adults (age ≥16 years) who are anxious about everyday situations because of agoraphobic-type fears and it is intended to reduce anxiety around other people. The software was programmed by Oxford VR/RealisedCare. The software is built using Unity (Unity Technologies). Unity acts as a render engine, displaying the virtual environments to the user through the headset. The application runs through the Unity software application on a Meta Quest 3S VR Headset. The programme is free-standing (ie, not dependent on NHS IT infrastructure and it does not connect with NHS electronic records) and simply runs on the headset.

The staff member in the first session can help show the person how to put on the headset and run the application. Detailed guidance on use is included within the application. Participants have a short tutorial showing them how to interact with the virtual environment and advising them of safety precautions. The application is supported by a staff member, who will be present for at least the first session but up to approximately six sessions (depending on patient preference). Additional sessions can be conducted remotely, especially if a participant has taken a headset home. The treatment is designed to be delivered in approximately six sessions, each involving 30 min in VR. It can be supported by a variety of mental health workers—the choice in each site will be made according to how it would be implemented in day-to-day practice. The staff member encourages the person to apply the learning from VR in the real world via helping set homework tasks to be carried out between sessions. The O-AS is also used by the staff member at meetings to monitor progress (and may be used later in research publications eg,to discover trajectories of change). The staff deliverer may also use the Oxford Cognitions and Defences Questionnaire to help guide treatment.^15^

gameChange is a cognitive treatment that aims for patients to relearn safety by testing their fear expectations. Within the VR environments, a virtual coach guides the person through the treatment. When first entering VR, the patient goes into the coach’s virtual office and is guided in how to use VR. At the beginning of the first session, the virtual coach explains the rationale behind the treatment, and the participant selects one of the six VR scenarios. The six VR scenarios are a: café, GP waiting room, pub, bus, street scene and newsagent. Each scenario has five degrees of difficulty (eg, the number and proximity of people in the social situation increases) and participants work their way through each level of difficulty. There are game type tasks within a number of the levels. A participant can choose a different scenario in each session or repeat a previous situation. gameChange is now delivered on standalone headsets. Headsets can be left with patients during the provision of the therapy, meaning that staff contact time could be lower and can also be largely done remotely. Patients may be encouraged to use the VR at home for 20–30 min at times that are most helpful for them.

### Control condition

Participants who are allocated to the waitlist control arm will continue to receive their usual care (TAU). TAU for the participants within this trial will typically consist of prescription of psychiatric medications and meetings with a mental health practitioner. TAU will vary across individuals. We will collect detailed data on TAU. After the 26-week assessment, gameChange will be provided to individuals in the control arm.

### Serious adverse events

SAEs are recorded using an SAE report form. Each participant’s medical notes will be systematically checked for SAEs following completion of the final assessment to ensure all SAEs are recorded. We will also record any SAEs that come to the attention of the assessor or gameChange deliverer. An adverse event is defined by the ISO14155:2011 guidelines for medical device trials as serious if it: (A) results in death, (B) is a life-threatening illness or injury, (C) requires hospitalisation or prolongation of existing hospitalisation, (D) results in persistent or significant disability or incapacity, (E) medical or surgical intervention is required to prevent any of the above, (F) leads to fetal distress, fetal death or consists of a congenital anomaly or birth defect or (G) is otherwise considered medically significant by the investigator. Life-threatening in the definition of an SAE refers to an event in which the subject was at risk of death at the time of the event; it does not refer to an event that hypothetically might have caused death if it were more severe. A planned hospitalisation for a pre-existing condition, without a serious deterioration in health, is not considered to be an SAE.

It is relatively common for this patient group to have SAEs, typically psychiatric hospital admissions, physical health hospital admissions and suicide attempts.

The relationship between gameChange or other research procedure and the occurrence of each SAE will be assessed and categorised. The chief investigator will use clinical judgement to determine the relationship. Alternative causes, such as natural history of the participant’s underlying condition, concomitant therapy, other risk factors, etc, will be considered. The investigator will also consult the current version of the risk analysis report. The chief investigator will make an initial assessment of whether the SAE is potentially related to the device or trial procedures, and the expectedness, and report as necessary to the regulatory authorities within the appropriate timescales (eg, related and unexpected SAEs to the research ethics committee and adverse incidents to the Medicines and Healthcare products Regulatory Agency). The decisions about relatedness and expectedness will be reviewed by the DMEC chair (an independent clinician) in the first instance, and later taken to a DMEC meeting.

We will also record adverse events that are not serious. This would include any adverse device effects from the VR programme, including those resulting from insufficient or inadequate instructions for use, deployment, installation or operation, or any malfunction of the software. It also includes any event resulting from user error or intentional misuse.

### Data management

All trial data will be entered on paper or electronic clinical research forms (CRFs) and transcribed or entered directly into the clinical data management system. The online Red Pill Electronic Data Capture system will be used (https://www.sealedenvelope.com/redpill/). Direct data entry by patients is also possible via Red Pill’s electronic patient-reported outcomes system. Data are pseudonymised using a unique study ID. Personal data and participant identification codes are kept separately from the research data. Access to these data is strictly on a need to know basis. Data are transferred from paper CRFs to the clinical database or recorded directly on eCRFs as soon as possible after a study visit. Validation of all data entered into the clinical database is achieved through manual review. All critical data items will be 100% checked against original source documents, where applicable.

### Analysis

A full statistical analysis plan will be approved before any analysis. We will report data in line with the CONSORT 2018–SPI^16^ and 2025 statements^17^ showing attrition rates and loss to follow-up. All outcome analyses will be conducted by statisticians at Oxford’s Primary Care Clinical Trials Unit. The target estimand is the treatment policy estimand and all primary and secondary analyses will be carried out incorporating data from all participants including those who do not complete treatment. Every effort will be made to follow up all participants in both arms for research assessments.

Treatment effects on primary and secondary outcomes will be estimated using linear mixed effect models fitted to outcome variables at both main follow-up points (8 and 26 weeks). Fixed effects will be site, baseline assessment for the outcome under investigation, treatment, time and time*treatment interactions. Participants will be included as a random intercept to account for repeated measures and nested within therapist to account for clustering by staff deliverer. Treatment effects will be estimated for the primary outcome at each time point and reported separately as adjusted mean differences in scores between the groups with 95% CIs and two-sided p values. We will also calculate an effect size (Cohen’s d), which will be the between group treatment difference divided by the shared SD at baseline.

We will check for differential predictors of missing outcomes by comparing responders to non-responders on key baseline variables. Any significant predictors will be included in the analysis models in a sensitivity analysis. This accounts for missing outcome data under a missing at random assumption, conditional on the covariates included in the model. As a sensitivity analysis, we will assess whether treatment adherence is associated with missing data, and if it is associated, use inverse probability weights or multiple imputation to compare results.

To test the moderation hypotheses, we will extend the analysis model to include as fixed effects the putative moderator and its interaction with treatment; the coefficient of the interaction tests whether there is a differential treatment effect across levels of the moderator variable.

Collection of 34-week data for the control group will provide further information for secondary trial reports on, for example, treatment uptake, satisfaction, acceptability, adverse effects and outcomes.

### Health economic analysis

A separate health economic analysis plan will be written in accordance with best practice for economic evaluations alongside a clinical trial. We will conduct a within-trial cost–utility analysis following the intention-to-treat principle and consider two perspectives: (1) NHS and Personal Social Services (NHS&PSS) and (2) societal (incorporating NHS&PSS and wider costs). The primary outcome measure of the economic evaluation will be incremental cost per QALY. The EQ-5D-5L and ReQoL collected at baseline, 8 weeks and 26 weeks will provide the utility values for the calculation of QALYs. We will estimate the costs of providing gameChange using a micro-costing approach. We will consider and measure costs such as training, travel, therapy delivery time, supervision time, hardware, software licence, maintenance, assistance from technical support staff as well as additional consumables and capital expenditures required. An adapted version of the CSRI^7 14^ will be used to collect data on healthcare and social care use, impact on work, criminal justice services and informal care received. Furthermore, information on admissions to mental health inpatient wards and medication prescribed will be obtained by reviewing medical records.

We will report descriptive statistics for resource use, costs and health utilities at each follow-up time point. Differences between arms will be estimated using multi-level mixed effects linear regression models to allow for multiple follow-ups clustered by participant.

We will follow best practice methods for addressing missing data in cost-effectiveness studies. The joint uncertainty around incremental total costs and QALYs will be estimated using seemingly unrelated regression and decision uncertainty will be represented using a cost-effectiveness plane.

### Power calculation

The number of participants is based on the means and SDs observed in our original trial. We assume an average cluster size of five participants per therapist and an ICC of 0.01. We calculate power both for the clinical outcome (agoraphobic avoidance) and quality of life.

O-AS-Avoidance: Based on a sample size of 200 and end of treatment means of 3.6 (SD=2.8) in the intervention group and 5.3 (SD=2.2) in the control group, we would have 90% power to detect a difference (5% significance). At 6 months, with means of 3.2 (SD=3.1) in the intervention group and 5.4 (SD=2.1) in the control group, we would have 99% power to detect a difference.

ReQol: Based on a sample size of 200 and end of treatment means of 37.5 (SD=18.7) in the intervention group and 30.6 (SD=13.2) in the control group, we would have 84% power to detect a difference (5% significance). At 6 months, with means of 38.1 (SD=18.0) in the intervention group and 31.3 (SD=12.5) in the control group, we would have 86% power to detect a difference.

## Ethics and dissemination

The trial has received Health Research Authority and Health and Care Research Wales (HCRW) approval (IRAS 351247). The trial received ethical approval from the HCRW Wales Research Ethics Committee 3 (25/WA/0081). The trial has not yet started recruiting participants. Recruitment is planned to start in July 2025, and for the trial to be completed by February 2028. Any changes to the trial protocol will have sponsor and ethical approvals. The results of the trial will be published in a peer-reviewed journal and made open access. Deidentified participant data will be available in anonymised form on reasonable request, subject to review and contract with the University of Oxford, following the publication of results.

### Patient and public involvement

The trial team has an individual with lived experience leading patient and public involvement. Two patient advisory group meetings, involving nine patients across the country, were held to discuss this evidence generation plan. There has also been considerable lived experience contributions to the development of the VR intervention. 53 people with lived experience contributed over 500 hours’ worth of input into the development of gameChange.^18 19^ The new trial is supported by a LEAP, who will meet regularly during the project.

## Supplementary material

10.1136/bmjopen-2025-104636 online supplemental material 1

10.1136/bmjopen-2025-104636 online supplemental material 2
