# Supplementary material for: Real-world waitlist randomised controlled trial of gameChange VR to treat severe agoraphobic avoidance in patients with psychosis: a study protocol
Source: BMJ Open. 2025 Aug 16;15(8):e104636. doi: 10.1136/bmjopen-2025-104636 (PMC12359439; doi:10.1136/bmjopen-2025-104636)
Supplement: online supplemental material 1 [file bmjopen-15-8-s001.docx]

**Consent Form: gameChange VR**

Participant ID:

*If you agree, please initial each box*

| 1. I confirm that I have read and understand the information sheet dated [insert date and version number] for this study. I have had the opportunity to consider the information, ask questions, and have had these answered satisfactorily. |  |
| --- | --- |
| 2. I understand that my participation is voluntary and that I am free to withdraw at any time without giving any reason, without my medical care or legal rights being affected. |  |
| 3. I understand that relevant sections of my medical notes and data collected during the study may be looked at by authorised individuals from University of Oxford, from regulatory authorities, and from NHS Trusts, where it is relevant to my taking part in this research. I give permission for these individuals to have access to my records. |  |
| 4. I understand that information I give to the study will be kept confidential unless I disclose significant risk to myself or other people, when my clinical team will need to be informed. |  |
| 5. I agree to my healthcare team being informed of my participation in the study. |  |
| 6. I agree to take part in this study. |  |

Name of participant: ______________________________

Signature: _______________________________________ ________ Date: ___ /___ /___

Name of person taking consent: ______________________________ Date: ___ /___ /___

Signature: ______________________________ Job title: _______________________

Tick if completed verbally and documented by person taking consent.
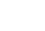


I would / would not (please delete as appropriate) like to receive a copy of the research findings

Preferred method of receiving the research findings if applicable: **email or post**
 **1 copy for participant; 1 (original) for researcher site file; 1 (scanned copy of original) to be uploaded to electronic medical notes*
