# Supplementary material for: Real-world waitlist randomised controlled trial of gameChange VR to treat severe agoraphobic avoidance in patients with psychosis: a study protocol
Source: BMJ Open. 2025 Aug 16;15(8):e104636. doi: 10.1136/bmjopen-2025-104636 (PMC12359439; doi:10.1136/bmjopen-2025-104636)
Supplement: online supplemental material 2 [file bmjopen-15-8-s002.docx]

**
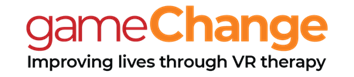
**

**Participant Information Sheet: gameChange VR**

We would like to invite you to take part in a study of virtual reality (VR) therapy. Before you decide, we would like you to understand why the research is being done and what it would involve for you. One of our team will go through the information sheet with you and answer any questions you have. Please ask us if anything is unclear or you would like more information.


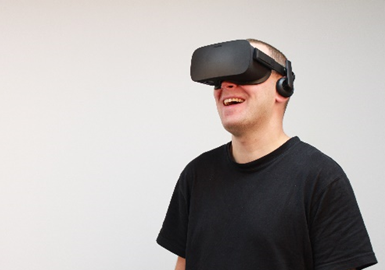

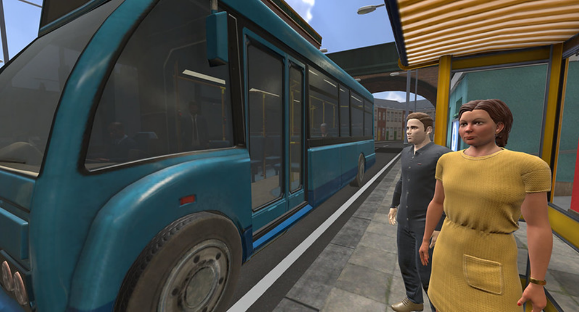


*A Virtual Reality (VR) headset and VR environment*

# Key facts:

- gameChange is for people attending mental health services for the treatment of psychosis who are finding it difficult to enter everyday situations. For example, it may be difficult to leave the home, or walk down the street alone, or get on a bus.
- gameChange virtual reality (VR) therapy is an easy to use, step–by-step programme. In VR simulations a person can practise, at their own pace, being in situations such as a café, a shop, or walking down the street. It was developed with people who have been through these difficulties. The idea is that practising in computer simulations can make the real situations easier too. The therapy is supported over eight weeks by a mental health staff member. A VR headset will be provided.
- gameChange is approved for use in the NHS for people with strong anxiety about going into everyday situations. The purpose of this new study is to provide further information of whether gameChange is helpful for individuals to overcome their anxiety and to work out the potential costs to the NHS.
- Everyone taking part will get the gameChange VR therapy. However, half of the people taking part will get it immediately and half will get it after six months. Whether a person has gameChange now or in six months is decided by chance. You will have a 50% chance of being in either group. We then compare how people who have had gameChange now have got on compared to people who have not had it yet.
- To see how everyone gets on, there will be a brief assessment at the beginning, which is then repeated after 8 and 26 weeks. For those people who have gameChange later there will also be an extra assessment after the therapy has been completed. You will be compensated for your time completing these assessments.
- It is entirely your choice whether to take part in the research or not. Your usual treatment will not be affected in any way by your decision and you will continue to have access to the standard of care you currently have. Even if you decide to take part, you will be free to leave at any time, for any reason.

# What is the purpose of the gameChange VR trial?

Many people have fears about everyday situations, for example walking down the street, going into a shop, getting on a bus, or being in a cafe. People may fear something bad will happen, for example that they will look foolish, or that they cannot cope, or that people will laugh or attack them in some way. The fears mean that people try to avoid these situations. This type of anxiety can be called ‘agoraphobia’.

gameChange is a promising virtual reality (VR) therapy for treating this anxiety. People practise overcoming their fear in computer simulations in order to make the real situations easier. It has already been tested in one study with approximately 350 patients. The results led to gameChange obtaining initial approval for use in the NHS. However further information is required for it to become more widely used in the future. The clinical benefits need to be shown again and the potential costs of providing it in the NHS calculated. That is, we need to check whether gameChange is potentially helpful for people and whether it is affordable for the NHS. This is the purpose of the gameChange VR trial.

# What is gameChange VR therapy?

VR is a computer simulation. It is a bit like a video game. By putting on a headset, a person sees, hears, and can move around the computer simulation. gameChange was developed with people who have had fears about everyday situations. The gameChange simulations are a café, shop, pub, street, doctor’s surgery, and a bus. The simulations allow a person to practise, at their own pace, being in everyday situations. A virtual coach (called Nic) guides the person through the VR therapy. Research has shown that overcoming fears in VR tends to lead to fewer fears in the real situations. The therapy is provided for eight weeks, with regular check-ins with a mental health professional. The VR headset is provided. The mental health professional will show you how to use the VR programme. Your usual NHS care will continue as before.


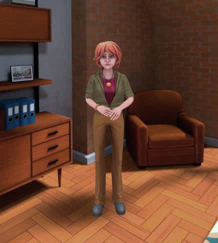


*Nic is a virtual coach who guides the person through the VR therapy*

# Who can take part in the research?

The research is for adults (16 years+) who feel very fearful and anxious about entering everyday situations, and are attending mental health services for the treatment of psychosis.

We will first check with you whether the research study is suitable for you. Our aim is for 200 people to take part in the research.

# Do I have to take part?

No. If you are eligible to take part, it is up to you to decide whether to take part. If you agree to take part we will then seek your consent either via signing a form or verbally over the telephone. . You are free to withdraw at any time, without giving a reason. Your usual treatment will not be affected if you withdraw from this study.

# What will happen if I take part?

Everyone who takes part will be asked to complete an initial assessment, which will take about half an hour. This asks about anxiety and fears, your wellbeing, and your use of health and social care services. The assessment can take place online, or at your local mental health clinic, or we may be able to meet at your home if you would prefer.

You will then either have the gameChange VR therapy straightaway or after six months. Whether you have gameChange immediately or after six months is decided by a computer (rather like flipping a coin). You will have a 50% chance of being in either group. After completing gameChange we will ask you to complete two questionnaires about the experience, concerning how acceptable you found it and whether you had any side effects. The initial assessment is then repeated after 8 weeks and after 26 weeks. If you have gameChange after six months then we will also repeat the assessment after you have had the therapy.

Therefore there will be three times the assessments are completed for people who receive gameChange immediately and four assessments for people who receive gameChange after six months. We get in contact with people when the assessments are due.

For more information on what participation in the study would look like, please see the diagram below:

Participation lasts either 6 or 8 months in total

As part of the study, we would also want to look at your medical notes. The staff member who helps support gameChange will need to do this as part of standard good care in the NHS. The study team will look at your notes to see how you are getting along and the care you receive. They will also record information on the services and support you receive from your medical notes. All of this will be done on a confidential basis.

# Expenses and payment

You will receive a [£10 payment by bank transfer/shopping voucher if applicable for local NHS Trust] for each of the assessment sessions you take part in. We will also reimburse you for any reasonable travel costs for attending an assessment. You will also be asked to sign a receipt to indicate you have received your [£10 payment by bank transfer/shopping voucher if applicable for local NHS Trust].

# What are the possible risks of taking part?

We do not anticipate any major risks from taking part. We have designed and tested the VR therapy to minimise any risks. We will check this with you during and after the VR therapy. It can occasionally happen that some people experience a degree of nausea (like car travel sickness) after using VR. Our testing over the years has not revealed this as a significant problem. If you have a history of photosensitive epilepsy then the study would not be right for you, since with any type of video equipment there is a possibility that an episode may occur. When using gameChange people will be presented with computer simulations of situations that typically make them anxious. Therefore it is likely you will feel a degree of anxiety when using gameChange. This is so that you can learn that you can cope and that nothing bad will happen and hence the anxiety will lessen. This situations are presented gradually and in an order that you choose, so that the anxiety should not become too much for you. There is also the gameChange mental health staff member to help support you during this process.

# What are the possible benefits of taking part?

Given our previous testing of gameChange we expect it should help people get back into some of the situations that they fear and reduce the fears and anxiety when in them. The research aims to find out whether this is the case.

# Will my taking part in the study be kept confidential?

The study team will keep your name, NHS number, and contact details confidential. To protect your identity, we will not use your name on any of the study documents. Instead of your name, we will assign you a unique code so that your information is kept private.

Your clinical team will be told you are taking part in the trial. All other information from the research assessments will be kept confidential unless you would like your clinical team to know. The exceptions to this are where there are significant concerns about a risk to you or other people. If a member of our team has such concerns we will need to tell your clinical team.

Responsible members of the University of Oxford, regulatory authorities, and the relevant NHS trust, may be given access to data for monitoring and/or audit of the study to ensure that the research is complying with applicable regulations.

# What will happen to my data?

Data protection regulation requires that we state the legal basis for processing information about you. In the case of research, this is ‘a task in the public interest.’ The University of Oxford, based in the UK, is the data controller and is responsible for looking after your information and using it properly. We will be using information from you and your medical records in order to carry out this study.

Information that can be used to identify you (e.g. your name or NHS number) helps the research team to keep in contact with you about the research study, make sure that relevant information about the study is recorded for your care, and ensures the quality of the study. During the research, the research team will need to use this type of information, but we will try to keep this to a minimum. We will not share this information with anyone outside the research team. We will keep information that might identify you for 3-6 months after the study has finished. This excludes any research documents with personal information, such as consent forms, which will be stored at the University for 10 years after the end of the study.

Your data will be stored securely with our research team at the local trial site and the University of Oxford. Any data that you provide on hard copies (i.e. pen and paper) will be kept in a locked filing cabinet at your local trial site. Any electronic data that is collected (e.g. emails, questionnaire scores) will be kept on a computer at your local trial site and the University of Oxford and will be protected with a password. Only members of the research team will know the password or have access to the filing cabinet. If you receive payments for the study via bank transfer then your bank details will be stored for seven years in accordance with University of Oxford financial policy.

Your NHS Trust will use your name, NHS number, home address, and contact details to contact you about the research study, and to oversee the quality of the study. They will keep identifiable information about you from this study in keeping with local policy for medical notes retention. A copy of the consent form from this study will be kept in your medical records for as long as those records are retained.

It is possible that in the future other researchers may be interested in carrying out further analysis of group data from the trial. They would only be given group data that has had identifying information about individuals removed (called de-identified data). This means that de-identified data could be shared and reused for scientific purposes to maximise what is learned from the research. Other researchers would not be given any information that could identify individuals.

Data protection regulation provides you with control over your personal data and how it is used. When you agree to your information being used in research, however, some of those rights may be limited in order for the research to be reliable and accurate. Further information about your wishes with respect to your personal data is available at https://compliance.web.ox.ac.uk/individual-rights or by contacting the University of Oxford’s Data Protection officer at data.protection@admin.ox.ac.uk. You can find out more about how we use your information by contacting the research team, using the contact details given at the end of this document.

# What will happen if I don't want to carry on with the study?

Participation is voluntary and you may change your mind at any stage. Withdrawing from the study will not affect the care you receive from the NHS. You can withdraw from the study, the assessments or both. If you decide you do not want us to use any of the data you have already provided, you will need to tell us within three months of originally agreeing to take part in the study otherwise we will not be able to withdraw your data from the trial analyses.

# What will happen to the results of this study?

The results of the study are written up, looking at the total effects of the treatment for all patients, and no one is identified. The aim is to use the results if they are positive to obtain a recommendation for gameChange to be used routinely in NHS mental health services. The work will also be presented in scientific papers and in conferences. We will make a summary of the results of the study available for you if you would like that. If you would like to see the results summary, we will keep your contact details so we can share this with you.

# Who is organising and funding the study?

The research is funded by the National Institute for Health and Care Research (NIHR), whose mission is to improve the health and wealth of the nation through research. It is also supported by the NIHR Oxford Health Biomedical Research Centre. The study is sponsored by the University of Oxford.

# What if there is a problem?

The investigators recognise the important contribution that volunteers make to medical research, and will make every effort to ensure your safety and wellbeing.

The University of Oxford, as sponsor, has appropriate insurance in place in the unlikely event that you suffer any harm as a direct consequence of your participation in this study. If something does go wrong, you are harmed during the research, and this is due to someone's negligence, then you may have grounds for a legal action for compensation. While the Sponsor will cooperate with any claim, you may wish to seek independent legal advice to ensure that you are properly represented in pursuing any complaint. The study doctor can advise you of further clinical action and refer you to a doctor within the NHS for treatment, if necessary’.

If you wish to complain about any aspect of the way in which you have been approached or treated, or how your information is handled during the course of this study, you should contact Professor Daniel Freeman (contact details below) or you may contact the University of Oxford Research Governance, Ethics & Assurance office (RGEA) by telephone 01865 616480 or email the director of RGEA at [rgea.complaints@admin.ox.ac.uk](mailto:rgea.complaints@admin.ox.ac.uk).

The Patient Advisory Liaison Service (PALS) is a confidential NHS service that can provide you with support for any complaints or queries you may have regarding the care you receive as an NHS patient. PALS is unable to provide information about this research study. If you wish to contact the PALS team please contact [insert relevant NHS site phone number and email from the PALS website]

**How have patients and the public been involved in this study?**

The gameChange VR therapy was developed with people who have experienced psychosis. This included being involved in the design process (e.g. choosing the scenarios) and trying out the VR situations. A group of people who have had such difficulties have also contributed to the development of this trial and will advise on how it is carried out. There is a person who has had such difficulties as a senior member of the research group.

# Who has reviewed the study?

All research in the NHS is looked at by an independent group of people, called a Research Ethics Committee, to protect participants’ interests. This study has been reviewed and given a favourable opinion by [insert REC name and reference].

* Please do not hesitate to ask us if you have any questions. It can also be helpful to talk to someone else about whether you’d like to take part *

# CONTACT DETAILS

If you have any questions about this study, please do not hesitate to contact us:

| [insert local RA details] | [insert local trial coordinator details] |
| --- | --- |
| [insert local site lead details] | Professor Daniel Freeman (Overall Trial Lead), University of Oxford and Oxford Health NHS Foundation Trust.  Email: [daniel.freeman@psy.ox.ac.uk](mailto:daniel.freeman@psy.ox.ac.uk)  Telephone number: 01865 613109 |
