# Supplementary material for: Real-world waitlist randomised controlled trial of gameChange VR to treat severe agoraphobic avoidance in patients with psychosis: a study protocol
Source: BMJ Open. 2025 Aug 16;15(8):e104636. doi: 10.1136/bmjopen-2025-104636 (PMC12359439; doi:10.1136/bmjopen-2025-104636)
Supplement: Supplementary file 3 [file bmjopen-2025-104636.reviewer_comments.pdf]

# PEER REVIEW HISTORY

BMJ Open publishes all reviews undertaken for accepted manuscripts. Reviewers are asked to complete a checklist review form and are provided with free text boxes to elaborate on their assessment. These free text comments are reproduced below.

**ARTICLE DETAILS**

**Title (Provisional)**

A Real-World Waitlist Randomised Controlled Trial of gameChange VR to treat severe agoraphobic avoidance in patients with psychosis: A Study Protocol

**Authors**

Freeman, Daniel; Jones, Julia; Prouten, Eloise; Sainsbury, John; Morrison, Anthony; Chapman, Kate; Cousins, Emma; Altoft, Victoria; Peel, Heather; Kabir, Thomas; Myrick, Jack; Rovira, Aitor; Rouse, Natalie; Waite, Felicity; Lambe, Sinéad; Leal, José; Yu, Ly-Mee

---

This article was not externally reviewed at BMJ Open. Protocols that have been independently assessed prior to submission to BMJ Open are usually fast-tracked to publication on the grounds that further substantial changes will not be possible. This independent assessment will usually be external, independent review for both a substantial grant award from a non-commercial or government funder and ethics approval.
